# Interlaminar Fracture Toughness Analysis for Reliability Improvement of Wind Turbine Blade Spar Elements Based on Pultruded Carbon Fiber-Reinforced Polymer Plate Manufacturing Method

**DOI:** 10.3390/ma18020357

**Published:** 2025-01-14

**Authors:** Hakgeun Kim, Yunjung Jang, Sejin Lee, Chanwoong Choi, Kiweon Kang

**Affiliations:** 1 The Innovation Research Center for Giant Wind Turbine System, Kunsan National University, Gunsan-si 54150, Republic of Korea; kimhakgeun37@gmail.com (H.K.);; 2 Jeonbuk Institute of Automotive Convergence Technology, Gunsan-si 54158, Republic of Korea; 3 Department of Mechanical Engineering, Kunsan National University, Gunsan-si 54150, Republic of Korea

**Keywords:** blade, composite material, interlaminar fracture toughness, pultrusion, wind turbine, Weibull distribution

## Abstract

The key structural components of a wind turbine blade, such as the skin, spar cap, and shear web, are fabricated from fiber-reinforced composite materials. The spar, predominantly manufactured via resin infusion—a process of resin injection and curing in carbon fibers—is prone to initial defects, such as pores, wrinkles, and delamination. This study suggests employing the pultrusion technique for spar production to consistently obtain a uniform cross-section and augment the reliability of both the manufacturing process and the design. In this context, this study introduces carbon fiber-reinforced polymer (CFRP/CFRP) and glass fiber-reinforced polymer (GFRP/CFRP) test specimens, which mimic the bonding structure of the spar cap, utilizing pultruded CFRP in accordance with ASTM standards to analyze the delamination traits of the spar. Delamination tests—covering Mode I (double cantilever beam), Mode II (end-notched flexure), and mixed mode (mixed-mode bending)—were performed to gauge displacement, load, and crack growth length. Through this crack growth mechanism, the interlaminar fracture toughness derived was examined, and the stiffness and strength changes compared to CFRP based on the existing prepreg manufacturing method were analyzed. In addition, the interlaminar fracture toughness for GFRP, which is a material in contact with the spar structure, was analyzed, and through this, it was confirmed that the crack behavior has less deviation compared to a single CFRP material depending on the stiffness difference between the materials when joining dissimilar materials. This means that the higher the elasticity of the high-stiffness material, the higher the initial crack resistance, but the crack growth behavior shows non-uniform characteristics thereafter. This comparison provides information for predicting interlaminar delamination damage within the interior and bonding area of the spar and skin and provides insight for securing the reliability of the design life.

## 1. Introduction

Wind power generation is experiencing a surge in demand, prompting the scaling up of turbines to augment the power output. This upscaling necessitates longer blades, which are crucial components of the turbine system [1,2,3]. These blades are constructed from various components, such as the skin, spar cap, and shear web, utilizing fiber-reinforced composite materials such as carbon fiber-reinforced plastic (CFRP) and glass fiber-reinforced plastic (GFRP) [4].

Composite materials, composed of two or more substances, can be manufactured via various methods. These include infusion, where the material is placed in a mold, vacuumed, and then cured following resin injection; filament winding, which involves impregnating continuous fibers with resin, winding these fibers around a cylindrical mold, and then curing; and pultrusion, where continuous fibers are impregnated with resin and pulled through a mold to produce a long, consistent cross-section [5]. The spar cap, a critical structure that supports the load of the blade and is bonded to the skin, is predominantly fabricated using carbon fiber-reinforced composite materials through the infusion process [6]. However, the process is prone to initial damage, such as pores, wrinkles, and delamination [7,8]. The production of spar caps via the pultrusion technique is advocated to enhance the reliability of both the manufacturing process and the design. It ensures a uniform and consistent cross-section, endowing spar caps with superior mechanical properties in the fiber direction and minimizing manufacturing defects. Consequently, this enhances the efficiency and lifespan of wind turbines.

Nevertheless, challenges arise in manufacturing spar caps owing to their considerable lengths and complex shapes, tailored to airfoil designs. These challenges include difficulties in adjusting bonding during manufacturing, potentially resulting in delamination defects. Moreover, initial damages and subsequent defects during the bonding process of the wind turbine blade components can escalate during operation, posing safety risks. This necessitates a detailed examination of damage mechanisms. And by virtue of the constant cross-section of pultrusions, blades were more flexible for the same amount of material compared to conventional blades because the structure could not be tapered. This led to much more aeroelastically active blades, which, in turn, led to operational problems [9]. In particular, ultra-large blades of 8 MW or more require the application of carbon composite materials to the spar cap, but the thickness is 90 to 100 mm, so there is a problem in forming with a thickness of 50 mm or more due to problems such as heat generation during curing when forming with existing prepreg materials, and there is also the problem of excessive material costs.

Specifically, the spar cap, which comprises composite materials, requires an evaluation of mechanical properties pertaining to the delamination defects. The deformation and stress of each layer under force vary based on the characteristics of the material. Previous research has focused on ensuring structural reliability and integrity by integrating composite materials into structural designs, supplemented by experimental and analytical analyses [10,11,12,13,14] and probabilistic assessments [15,16,17] to determine the structural lifespan. However, studies that specifically address the delamination in materials layered via the pultrusion process remain limited [18,19].

**Figure 1:**
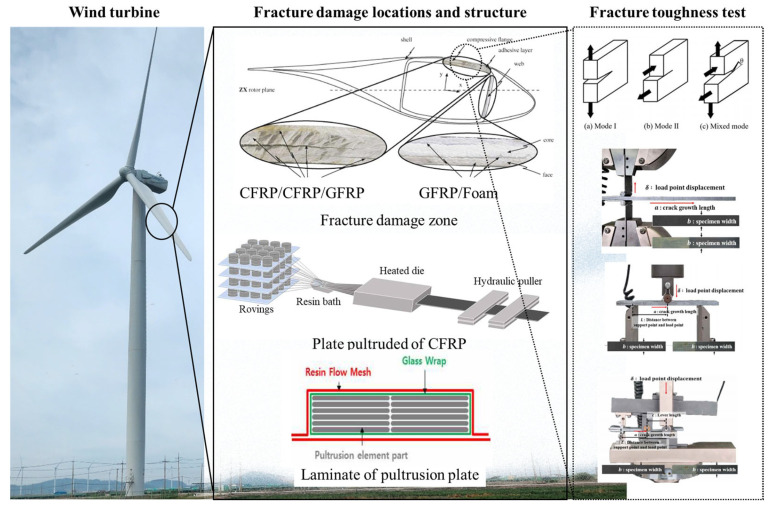
Application and analysis method of pultruded CFRP for wind turbine blades.

This study aims to quantify the properties of delamination defects of spar caps fabricated using the pultrusion method, particularly in the internal and bonding areas of the spar cap and skin, as shown in Figure 1. To this end, test specimens that conform to ASTM standards [20,21,22] were created using carbon fiber-reinforced composite materials for spar caps and glass fiber-reinforced composite materials for skins, both produced via pultrusion. The evaluation focused on the delamination characteristics of wind turbine blade spar caps using two types of test specimens: carbon fiber-reinforced polymer (CFRP/CFRP) and glass fiber-reinforced polymer (GFRP/CFRP), which replicate the bonding structure of the spar cap. The investigation covered different delamination modes, including Mode I, Mode II, and mixed-mode tests.

## 2. Test Methods of Fracture Toughness

### 2.1. Material and Specimen

**Figure 2:**
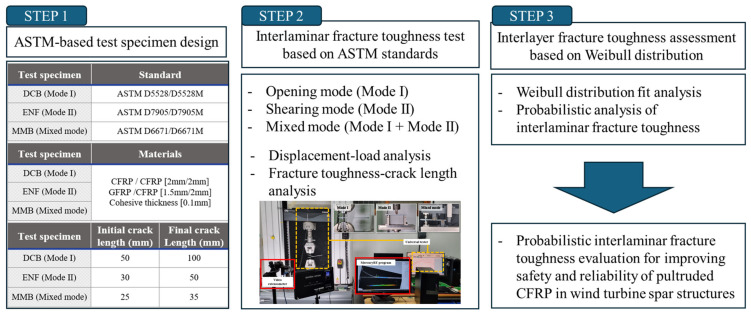
Flow chart for probabilistic interlaminar fracture toughness analysis.

**Figure 3:**
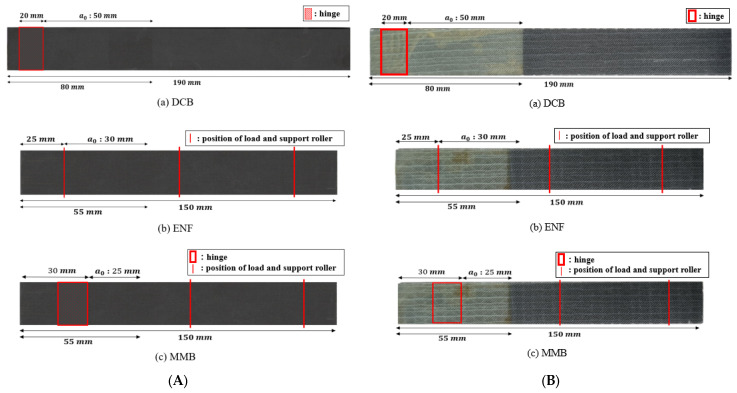
Dimensions of test specimens: (**A**) CFRP/CFRP, (**B**) GFRP/CFRP.

This study focuses on designing test specimens that replicate the structure of a spar cap, which is vital for maintaining the structural integrity of wind turbine blades, using the pultrusion process as shown in Figure 2. CFRP and GFRP were chosen as the materials for this study. For CFRP, a 2 mm thick composite material produced through pultrusion was used. The GFRP employed a laminated orientation of [±45°/0°] with a thickness of [0.6 mm/0.9 mm]. Test specimens were designed to simulate the internal bonding of the spar cap as CFRP/CFRP and the bonding area between the skin and the spar cap as GFRP/CFRP. The nomenclature for the delamination test specimens is delineated as follows: Mode I (opening test) is represented by a double cantilever beam (DCB), Mode II (three-point bending test) by an end-notched flexure (ENF), and mixed mode (combining Mode I and Mode II tests) by mixed-mode bending (MMB). Mode I specimens were fabricated with dimensions of 190 mm × 25 mm, adhering to ASTM D5528 [18]. Both Mode II and mixed-mode specimens were produced in the size of 150 mm × 25 mm, in line with ASTM D7905 [19] and ASTM D6671 [17] standards for ENF and MMB specimens, respectively. A 0.05 mm Teflon film was inserted between the layers of each specimen to facilitate crack growth, and the hinge attachment length and initial crack length were considered. This is to prevent more impregnation than the designed initial crack since the specimens were manufactured by the injection method. In particular, the initial crack lengths were designated as 50 mm for DCB, 30 mm for ENF, and 25 mm for MMB based on the ASTM standard to derive quantitative interlaminar fracture toughness. Figure 3 illustrates the configurations of the CFRP/CFRP and GFRP/CFRP delamination test specimens.

### 2.2. Delamination Test Method

**Figure 4:**
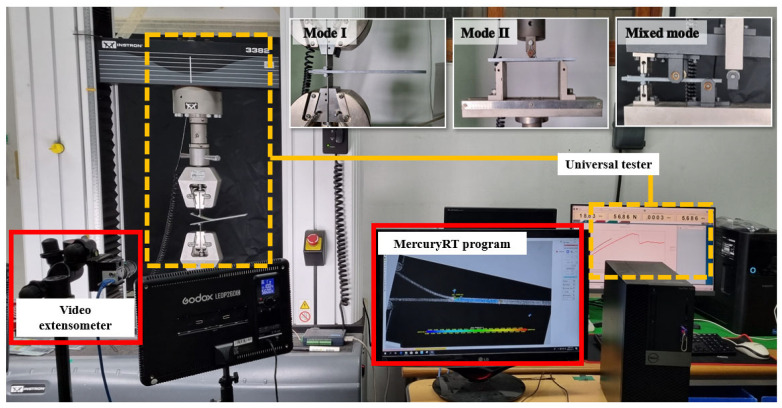
Experimental setup for crack growth testing.

The apparatus for the delamination test comprised an Instron 3382 universal testing machine (INSTRON, Norwood, MA, USA) and a video extensometer (iMETRUM Ltd., Bristol, UK), paired with the Mercury RT software, (iMETRUM Ltd., Bristol, UK) for monitoring crack growth. For experiments that require a hinge, such as Mode I and mixed mode, the hinges were pre-attached to the upper and lower sections of the specimen. For Mode II tests (three-point bending), support and load points were marked on the upper part of the specimen, as shown in Figure 4.

#### 2.2.1. Delamination Test Method of CFRP/CFRP

**Figure 5:**
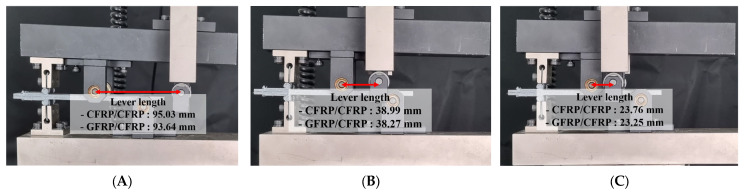
Lever lengths in mixed-mode testing: (**A**) Mixeture ratio 0.2, (**B**) 0.55, (**C**) 0.9.

In this study, Mode I, Mode II, and mixed-mode delamination tests were conducted to assess the fracture toughness in wind turbine blade components. In particular, the initial test for each mode test was a test that applied an initial crack tip within 5 mm to uniformly form the initial crack tip of the manufactured test specimen. Then, the main test was performed after unloading based on the ASTM standard. For Mode I, a pre-crack (PC) that forms the initial crack was generated within a crack growth length of 4 mm at a displacement control speed of 0.2 mm/min based on the standard. This was followed by unloading and then continuing at a speed of 0.8 mm/min, with crack growth monitored up to 50 mm. In Mode II, the test was performed at a displacement control speed of 1 mm/min, observing crack growth until it reached 20 mm. Mixed-mode testing, combining elements of Mode I and Mode II, was conducted to observe nonlinear crack growth behavior. Before the test, mode mixture ratios of 0.2, 0.55, and 0.9 were selected. These were selected according to the relationship $G_{II}/G_{I+II}$ based on the interlaminar fracture toughness characteristics of Mode I and Mode II. The closer this relationship is to 0, the more it has the characteristics of Mode I, and the closer it is to 1, the more it shows the characteristics of Mode II. And the test was carried out at a displacement control speed of 0.4 mm/min, with the crack growth length observed until 10 mm, as shown in Figure 5.

#### 2.2.2. Delamination Test Method of GFRP/CFRP

In Mode I, the test was executed at a displacement control speed of 1.0 mm/min, observing crack growth up to 50 mm. In Mode II, the test was conducted again at a displacement control speed of 1 mm/min, with crack growth observed until it reached 20 mm. For the mixed mode, the same mode mixture ratios were used, and the test was performed at a displacement control speed of 0.6 mm/min, monitoring crack growth up to 10 mm.

## 3. Test Results and Discussion

### 3.1. Test Results of CFRP/CFRP Fracture Toughness

#### 3.1.1. Mode I Test Result of CFRP/CFRP

The test conducted in Mode I revealed that the average load that corresponded to the hinge displacement was 146.9 N at a displacement of 9.08 mm. Employing the point at which delamination is visually observed on the edge (VIS) technique and based on the onset of crack growth, the critical fracture toughness was calculated as approximately 1.68 kJ/m^2^ using Equation (1).

(1) $$G_{Ic}=\frac{3P\delta }{2ba}$$

**Figure 6:**
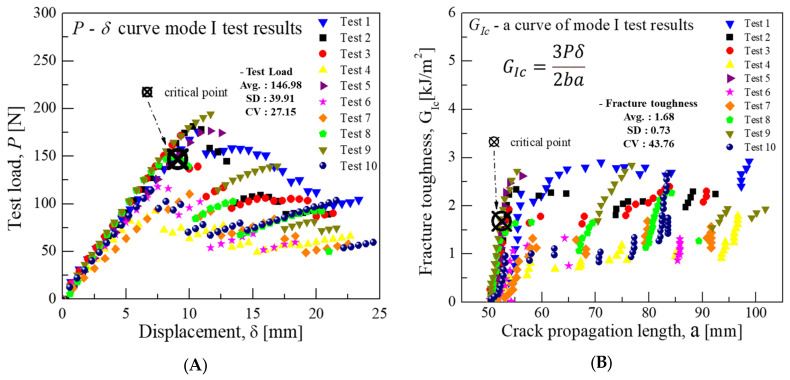
Mode I test results for fracture toughness of CFRP/CFRP: (**A**) Load-Disp. Curve, (**B**) Fracture toughness-Crack length curve.

In Equation (1), $G_{Ic}$ represents the critical energy release rate in Mode I, P denotes the critical load, δ symbolizes the critical displacement, b denotes the specimen width, and a denotes the crack growth length. The results of this analysis are depicted in Figure 6.

#### 3.1.2. Mode II Test Result of CFRP/CFRP

**Figure 7:**
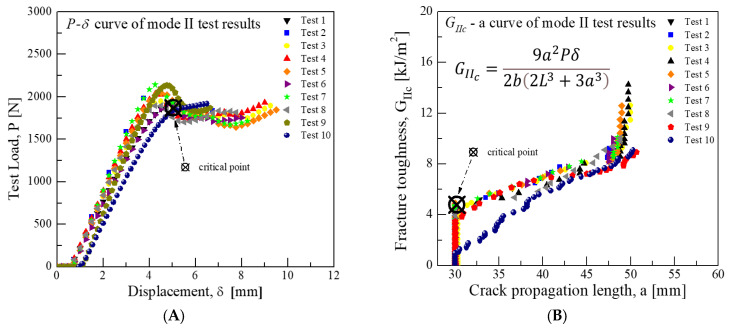
Mode II test results for fracture toughness of CFRP/CFRP: (**A**) Load-Disp. Curve, (**B**) Fracture toughness-Crack length curve.

During the Mode II test, a load of 1867.8 N was recorded at an average displacement of 5.04 mm. Using Equation (2) and considering the crack growth point as the critical point, the average critical fracture toughness was subsequently calculated to be approximately 4.79 kJ/m^2^. The results of this analysis are depicted in Figure 7.

(2) $$G_{IIc}=\frac{9a^{2}P\delta }{2b\left(2L^{3}+3a^{3}\right)}$$

#### 3.1.3. Mixed-Mode Test Result of CFRP/CFRP

In the mixed-mode test, which incorporates characteristics of both Mode I and Mode II, the average loads that correspond to the displacement at the load point were calculated for mode mixture ratios of 0.2, 0.55, and 0.9. The lever lengths used in the calculations were 95.03 mm, 38.99 mm, and 23.76 mm, respectively, as determined by Equations (3)–(7).

(3) $$\Gamma =1.18\frac{\sqrt{E_{11}E_{22}}}{G_{13}}$$

(4) $$\chi =\sqrt{\frac{E_{11}}{11G_{13}}\left\{3-2{\left(\frac{\Gamma }{1+\Gamma }\right)}^{2}\right\}}$$

(5) $$\alpha =\frac{1-\frac{G_{II}}{G_{I+II}}}{\frac{G_{II}}{G_{I+II}}}=\frac{G_{I+II}}{G_{II}}-1\;$$

(6) $$\beta =\frac{a+\chi h}{a+0.42\chi h}$$

(7) $$c=\frac{12\beta^{2}+3\alpha +8\beta \sqrt{3\alpha }}{36\beta^{2}-3\alpha }L$$

where Γ is the transverse modulus correction parameter, *χ* is the crack length correction parameter, *α* is the mode mixture transformation parameter for setting the lever length, and *β* is the non-dimensional crack length correction for the mode mixture.

Consequently, a displacement of 4.71 mm and a load of 152.3 N were obtained for a mode mixture ratio of 0.2. For a ratio of 0.55, the displacement was 3.18 mm with a load of 496.7 N, and for a ratio of 0.9, the displacement was 4.37 mm with a load of 1023.3 N. The critical interlaminar fracture toughness for the mixed mode was calculated using Equations (8)–(10). In accordance with the nonlinear (NL) criterion, the calculation involved analyzing points beyond the linear section of the load–displacement diagram, excluding the initial nonlinear section.

(8) $$G_{Ic}=\frac{12P^{2}{\left(3c-L\right)}^{2}}{16b^{2}h^{3}L^{2}E_{1f}}{\left(a+\chi h\right)}^{2}$$

(9) $$G_{IIc}=\frac{9P^{2}{\left(c+L\right)}^{2}}{16b^{2}h^{3}L^{2}E_{1f}}{\left(a+0.42\chi h\right)}^{2}.$$

(10) $$G_{\left(I+II\right)c}=G_{Ic}+G_{IIc}.\;$$

**Figure 8:**
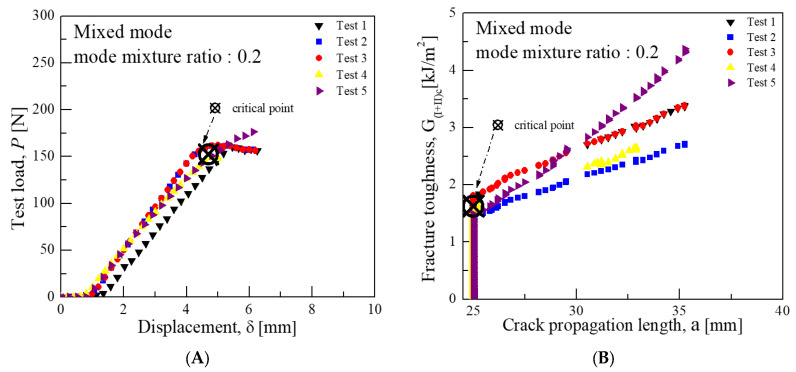
Mixed-mode test results for fracture toughness at mode mixture ratio 0.2 (CFRP/CFRP): (**A**) Load-Disp. Curve, (**B**) Fracture toughness-Crack length curve.

**Figure 9:**
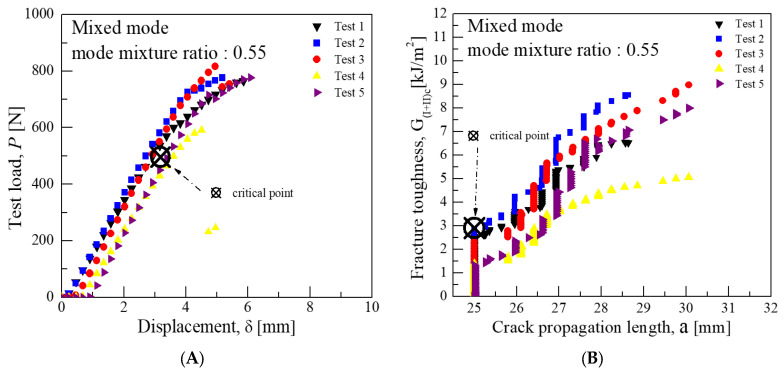
Mixed-mode test results for fracture toughness at mode mixture ratio 0.55 (CFRP/CFRP): (**A**) Load-Disp. Curve, (**B**) Fracture toughness-Crack length curve.

**Figure 10:**
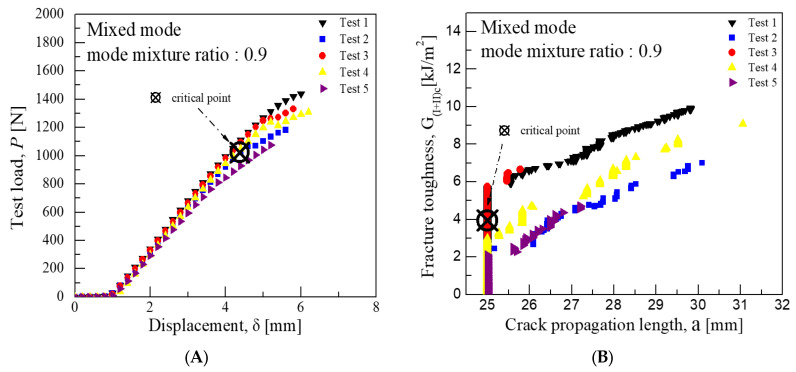
Mixed-mode test results for fracture toughness at a mode mixture ratio 0.9 (CFRP/CFRP): (**A**) Load-Disp. Curve, (**B**) Fracture toughness-Crack length curve.

Consequently, the average critical interlaminar fracture toughness was approximately 1.62 kJ/m^2^ for a mode mixture ratio of 0.2, 2.91 kJ/m^2^ for 0.55, and 3.94 kJ/m^2^ for 0.9. These values indicate that the closer the mode mixture is to Mode I characteristics, the more the interlaminar fracture toughness value aligns with that of Mode I, and similarly, the closer it is to Mode II characteristics, the more it aligns with the toughness value of Mode II. Figure 8, Figure 9 and Figure 10 illustrate the graphs of test results for each mode mixture ratio.

### 3.2. GFRP/CFRP Fracture Toughness Results

#### 3.2.1. Mode I Test Result of GFRP/CFRP

**Figure 11:**
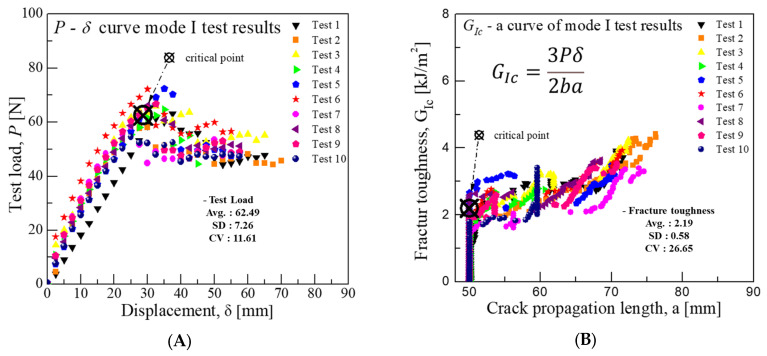
Mode I test results for fracture toughness of GFRP/CFRP: (**A**) Load-Disp. Curve, (**B**) Fracture toughness-Crack length curve.

In the Mode I test for GFRP/CFRP, an average displacement of 28.73 mm resulted in a load of 62.5 N. Utilizing the VIS technique and referencing Equation (1), the average critical fracture toughness was calculated to be approximately 2.19 kJ/m^2^. These results are shown in Figure 11. In particular, there is a large difference from the critical load point displacement of 9.08 mm for CFRP/CFRP mentioned in Figure 5. This is considered to be a phenomenon that occurs because the stiffness of the GFRP material is lower than that of the CFRP material.

#### 3.2.2. Mode II Test Result of GFRP/CFRP

**Figure 12:**
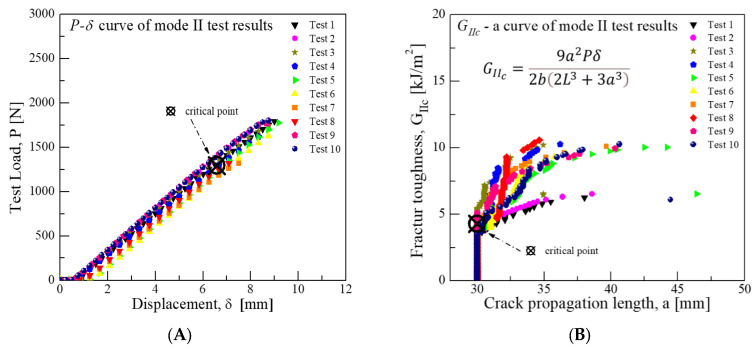
Mode II test results for fracture toughness of GFRP/CFRP: (**A**) Load-Disp. Curve, (**B**) Fracture toughness-Crack length curve.

During the Mode II test, a load of 1294.2 N was observed at an average displacement of 6.58 mm. The average critical fracture toughness, determined based on Equation (2) and considering the crack growth point as the critical point, was calculated to be approximately 4.28 kJ/m^2^. The experimental results for Mode II are displayed in Figure 12.

#### 3.2.3. Mixed-Mode Test Result of GFRP/CFRP

**Figure 13:**
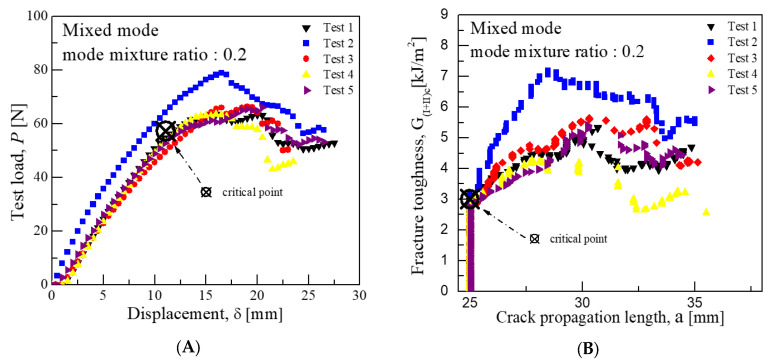
Mixed-mode test results for fracture toughness at mode mixture ratio 0.2 (GFRP/CFRP): (**A**) Load-Disp. Curve, (**B**) Fracture toughness-Crack length curve.

**Figure 14:**
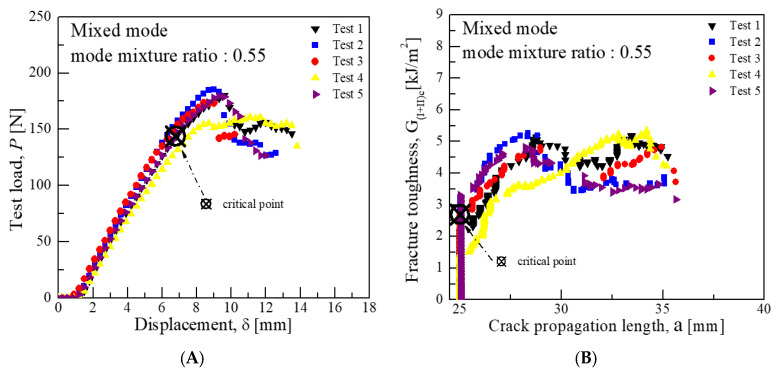
Mixed-mode test results for fracture toughness at mode mixture ratio 0.55 (GFRP/CFRP): (**A**) Load-Disp. Curve, (**B**) Fracture toughness-Crack length curve.

**Figure 15:**
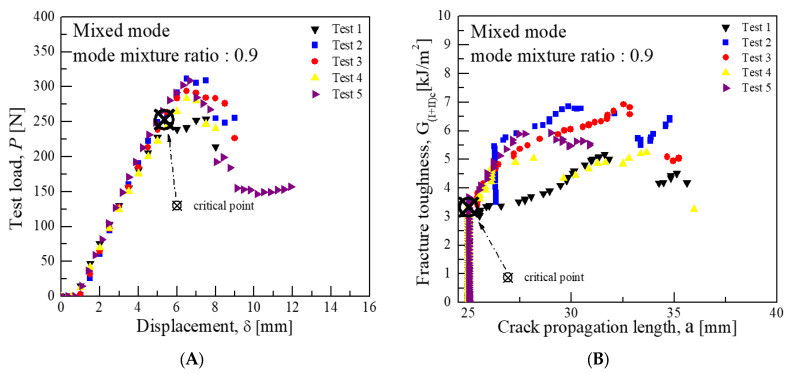
Mixed-mode test results for fracture toughness at mode mixture ratio 0.9 (GFRP/CFRP): (**A**) Load-Disp. Curve, (**B**) Fracture toughness-Crack length curve.

In the mixed-mode test, the lever lengths were calculated using Equations (3)–(7), yielding lengths of 93.64 mm for a mode mixture ratio of 0.2, as shown in Figure 13; 38.27 mm for 0.55, as shown in Figure 14; and 23.25 mm for 0.9, as shown in Figure 15. Subsequently, the average load values that correspond to the displacement at the load point were determined for these lever lengths. For a mode mixture ratio of 0.2, a displacement of 11.06 mm resulted in a load of 57.3 N; for 0.55, a displacement of 6.77 mm produced a load of 143.8 N; and for 0.9, a displacement of 5.34 mm resulted in a load of 253 N. The critical interlaminar fracture toughness for the mixed mode was calculated in accordance with Equations (8)–(10), adhering to the NL criterion as specified in Section 3.1.3. Consequently, the average critical interlaminar fracture toughness was determined to be 3.0 kJ/m^2^ for a mode mixture ratio of 0.2, 2.69 kJ/m^2^ for 0.55, and 3.34 kJ/m^2^ for 0.9, demonstrating distinct interlaminar fracture toughness values for each mode mixture ratio. The standard deviation results from Mode I, Mode II, and mixed-mode tests indicate similar patterns of crack growth at comparable displacements and loads.

### 3.3. Interlaminar Fracture Toughness Probabilistic Analysis

#### 3.3.1. Weibull Distribution Fit Evaluation for Interlaminar Fracture Toughness

**Figure 16:**
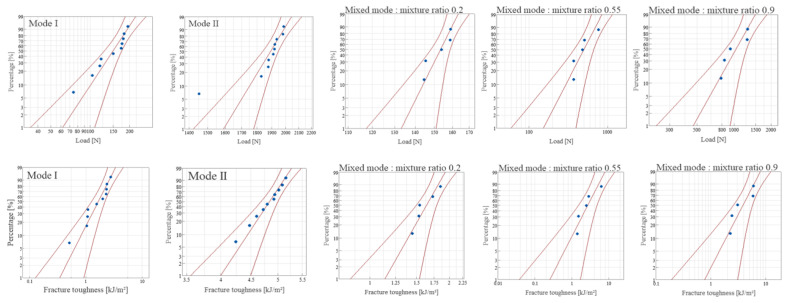
Weibull probability plot for fracture toughness of CFRP/CFRP.

**Figure 17:**
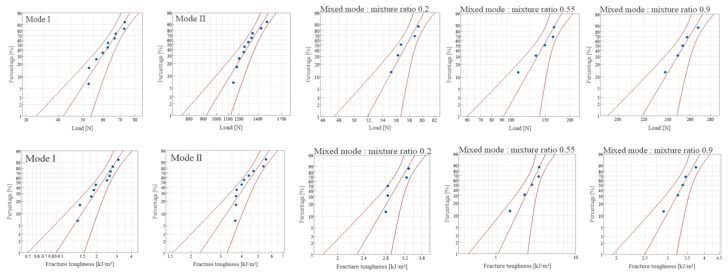
Weibull probability plot for fracture toughness of GFRP/CFRP.

A probabilistic analysis employing the Weibull distribution was performed to enhance the understanding of the experimental results regarding interlaminar fracture toughness. The suitability of the Weibull distribution for this purpose was assessed by plotting the load and interlaminar fracture toughness results on a Weibull probability plot. This plot, created using commercial Minitab [23] statistical analysis software at a 95% confidence level, is displayed in Figure 16 and Figure 17. The alignment of the results within the confidence interval and their close approximation to a straight line indicate the appropriateness of the Weibull distribution for these data. Following this, the Weibull distribution parameters were estimated using the maximum likelihood method based on the observed values, and these estimated parameters are listed in Table 1, Table 2, Table 3 and Table 4.

#### 3.3.2. Probabilistic Analysis of CFRP/CFRP Interlaminar Fracture Toughness

**Figure 18:**
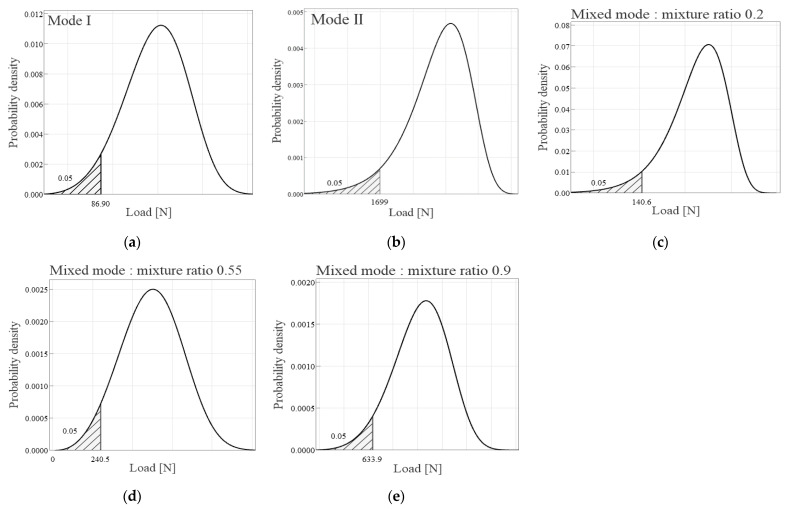
Probability density function for load of CFRP/CFRP: (**a**) *P_Ic_*, (**b**) *P_IIc_*, (**c**) *P*_(*I+II*)*c*_ for mixture ratio 0.2, (**d**) *P*_(*I+II*)*c*_ for mixture ratio 0.55, and (**e**) *P*_(*I+II*)*c*_ for mixture ratio 0.9.

To perform a clearer evaluation of the interlayer separation characteristics inside the spar cap, the probability density function is presented based on the parameters estimated in Section 3.3.2. This function facilitated a comparative analysis between the characteristic values derived from the normal distribution and the lower 5% values of the probability density function of the Weibull distribution, as shown in Figure 18. In this comparison, it was observed that the characteristic value *R_k_* for Mode I, with a load of 60.6 N and interlaminar fracture toughness of 0.09 kJ/m^2^, is more conservative than the corresponding lower 5% Weibull distribution value, as shown in Table 5. Similarly, for Mode II, the characteristic value $R_{k}$, featuring a load of 1535.5 N and interlaminar fracture toughness of 4.17 kJ/m^2^, was found to be more conservative compared to the Weibull distribution values. When assessing a mode mixture ratio of 0.2, the characteristic value $R_{k}$ with a load of 135.6 N and interlaminar fracture toughness of 1.23 kJ/m^2^ also proved to be more conservative than the Weibull distribution values. However, for mode mixture ratios of 0.55 and 0.9, large variances in the test results led to the calculation of negative values for interlaminar fracture toughness despite the evaluations at 114.9 N and 443.9 N, respectively. In the mixed mode, the variability of the material properties was large, which was stable at the mixing ratio of 0.2, but as the load difference changed rapidly as it approached 0.9, Mode II characteristics began to occur. Due to this, the phenomenon in which the load, which should increase from 0.2 to 0.9, actually decreases due to rapid crack growth caused by high energy generation in the single-material CFRP/CFRP was examined. This phenomenon occurs similarly in GFRP/CFRP, but the negative characteristics were not confirmed due to the relatively low load difference due to the GFRP material. In conclusion, the fracture characteristics in the mixed mode are judged to be relatively weaker than in the single-load mode.

#### 3.3.3. Probabilistic Analysis of GFRP/CFRP Interlaminar Fracture Toughness

**Figure 19:**
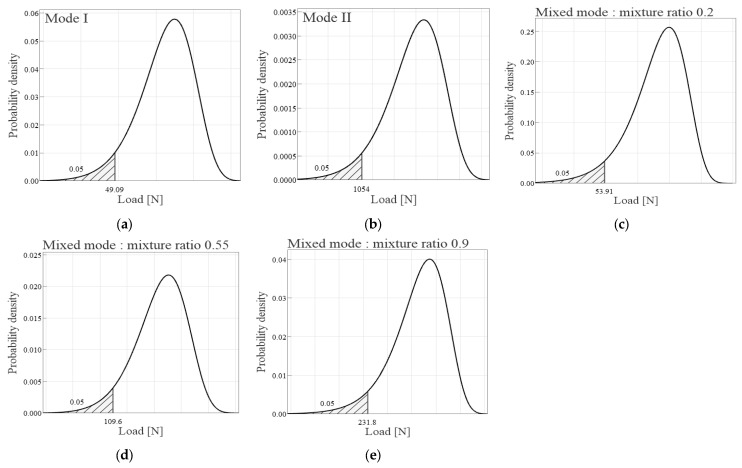
Probability density function for load of GFRP/CFRP: (**a**) *P_Ic_*, (**b**) *P_IIc_*, (**c**) *P*_(*I+II*)*c*_ for mixture ratio 0.2, (**d**) *P*_(*I+II*)*c*_ for mixture ratio 0.55, (**e**) *P*_(*I+II*)*c*_ for mixture ratio 0.9.

In the analysis of GFRP/CFRP for Mode I, a comparison between the characteristic value *R_k_* and the lower 5% value of the Weibull distribution indicated that $R_{k}$ is more conservative, as shown in Figure 19. Specifically, *R_k_* corresponded to a load of 60.6 N and an interlaminar fracture toughness of 0.09 kJ/m^2^, as shown in Table 6. For Mode II, the characteristic value *R_k_*, characterized by a load of 1535.5 N and an interlaminar fracture toughness of 4.17 kJ/m^2^, was found to be more conservative compared to the corresponding Weibull distribution values. Furthermore, when comparing characteristic values for a mode mixture ratio of 0.2, $R_{k}$, with a load of 135.6 N and interlaminar fracture toughness of 1.23 kJ/m^2^, was again more conservative than the Weibull distribution values. However, for mode mixture ratios of 0.55 and 0.9, the evaluations yielded loads of 114.9 N and 443.9 N, respectively. Owing to significant variances in the test results, the interlaminar fracture toughness values for these ratios were calculated to be negative. Overall, the comparison of characteristic values with the Weibull distribution outcomes demonstrated a more conservative trend for the characteristic values. This implies that the characteristic values provide a more cautious estimate of the fracture toughness compared to those obtained through the Weibull distribution analysis.

### 3.4. Performance Comparison Between Pultrusion and Prepreg Methods

CFRP manufactured using the pultrusion method demonstrated superior crack resistance and energy absorption compared to the conventional prepreg method. The pultrusion method offers consistent quality, high production speed, and suitability for large-scale structures, making it particularly advantageous for ultra-large applications, such as wind turbine blades. Conversely, the prepreg method exhibits higher initial stiffness, which can be beneficial in specific applications, but it is limited by slower processing speeds and higher production costs [24].

**Figure 20:**
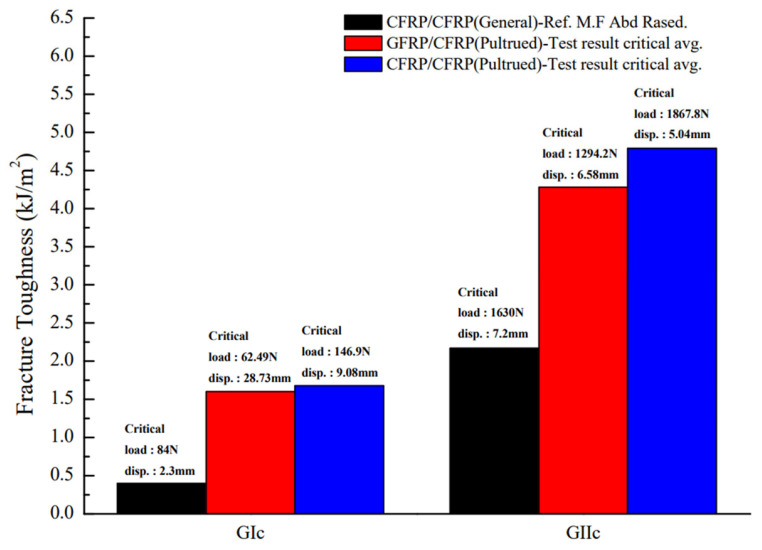
Comparative analysis of fracture toughness between prepreg and pultrusion methods.

Figure 20 compares the fracture toughness values between the pultrusion and prepreg methods. In Mode I, the prepreg method showed superior initial stiffness and structural rigidity. However, in mixed-mode and Mode II conditions, the pultrusion method outperformed by demonstrating enhanced energy absorption and crack resistance. Notably, CFRP produced through the pultrusion method effectively suppressed crack propagation under mixed-mode loading, maintaining stable performance. This indicates that the pultrusion method enhances structural reliability in large-scale applications.

In conclusion, the pultrusion method presents an economical and reliable alternative for the design and manufacturing of large-scale structures, such as ultra-large wind turbine blades. It offers the potential to reduce manufacturing costs while ensuring long-term performance.

## 4. Conclusions

This study focused on the delamination behavior of spar caps constructed from pultruded CFRP, emphasizing interlaminar fracture toughness through Weibull distribution analysis. Specimens were designed to replicate the internal structure and bonding interfaces of spar caps, and delamination tests were performed under Mode I, Mode II, and mixed-mode conditions. The relationships between displacement, load, and crack growth length were analyzed to determine fracture toughness for each mode.

The study employed probabilistic methods to assess fracture toughness variability. Weibull distribution analysis enabled the estimation of shape and scale parameters, providing deeper insights into the statistical characteristics of fracture behavior. Probability density functions and confidence intervals were established, highlighting the significance of load variability in spar cap design.

The findings revealed that pultruded CFRP spar caps exhibit superior crack resistance and mechanical performance, particularly under mixed-mode conditions, compared to traditional methods. This makes them highly suitable for ultra-large wind turbine blades. The integration of probabilistic analysis ensures enhanced structural reliability and safety while also optimizing design efficiency.

In conclusion, this research underscores the importance of combining advanced materials like pultruded CFRP with probabilistic methods to achieve cost-effective, reliable, and high-performance wind turbine components. These insights provide a foundation for future studies to improve material properties, manufacturing techniques, and design processes for large-scale applications.

## Figures and Tables

**Table 1:** Weibull parameters for CFRP/CFRP fracture toughness.

	Mode I	Mode II	Mixed Mode		
			0.2	0.55	0.9
α	2.38	22.22	11.58	1.78	2.6
λ (kJ/m^2^)	1.95	4.92	1.69	3.3	4.47

**Table 2:** Weibull parameters for GFRP/CFRP fracture toughness.

	Mode I	Mode II	Mixed Mode		
			0.2	0.55	0.9
α	4.58	6.49	15.18	4.44	14.09
λ (kJ/m^2^)	2.40	4.58	3.1	2.97	3.46

**Table 3:** Weibull parameters for CFRP/CFRP load.

	Mode I	Mode II	Mixed Mode		
			0.2	0.55	0.9
α	4.80	24.39	29.81	3.58	5.27
λ (N)	161.27	1918.51	155.30	550.94	1113.39

**Table 4:** Weibull parameters for GFRP/CFRP load.

	Mode I	Mode II	Mixed Mode		
			0.2	0.55	0.9
α	10.25	12.15	40.46	8.98	28.04
λ (N)	65.59	1345.39	58.02	152.56	257.72

**Table 5:** Comparison of characteristic values for CFRP/CFRP.

Layup	Mode	R_k_		Weibull Distribution CDF-5%	
		P (N)	Gc (kJ/m^2^)	P (N)	Gc (kJ/m^2^)
CFRP/CFRP	I	24.39	29.81	3.58	5.27
	II	1535.5	4.17	1699	4.30
	Mixed (0.2)	135.6	1.23	140.6	1.31
	Mixed (0.55)	114.9	−1.83	240.5	0.63
	Mixed (0.9)	443.9	−0.51	633.9	1.43

**Table 6:** Comparison of characteristic values for GFRP/CFRP.

Layup	Mode	R_k_		Weibull Distribution CDF-5%	
		P (N)	Gc (kJ/m^2^)	P (N)	Gc (kJ/m^2^)
GFRP/CFRP	I	46.8	0.93	49.1	1.26
	II	1050	2.76	1054	2.90
	Mixed (0.2)	53.1	2.43	53.9	2.55
	Mixed (0.55)	88.2	0.72	109.6	1.52
	Mixed (0.9)	226.5	2.61	231.8	2.81

## Data Availability

The original contributions presented in the study are included in the article, further inquiries can be directed to the corresponding author.
